# Focus on the Contribution of Oxidative Stress in Skin Aging

**DOI:** 10.3390/antiox11061121

**Published:** 2022-06-06

**Authors:** Federica Papaccio, Andrea D′Arino, Silvia Caputo, Barbara Bellei

**Affiliations:** Laboratory of Cutaneous Physiopathology and Integrated Center of Metabolomics Research, San Gallicano Dermatological Institute, IRCCS, 00144 Rome, Italy; federica.papaccio@ifo.it (F.P.); silvia.caputo@ifo.it (S.C.)

**Keywords:** skin, photoprotection, epidermis, dermis, inflammation, UV, melanocyte, skin cancer, aging

## Abstract

Skin aging is one of the most evident signs of human aging. Modification of the skin during the life span is characterized by fine lines and wrinkling, loss of elasticity and volume, laxity, rough-textured appearance, and pallor. In contrast, photoaged skin is associated with uneven pigmentation (age spot) and is markedly wrinkled. At the cellular and molecular level, it consists of multiple interconnected processes based on biochemical reactions, genetic programs, and occurrence of external stimulation. The principal cellular perturbation in the skin driving senescence is the alteration of oxidative balance. In chronological aging, reactive oxygen species (ROS) are produced mainly through cellular oxidative metabolism during adenosine triphosphate (ATP) generation from glucose and mitochondrial dysfunction, whereas in extrinsic aging, loss of redox equilibrium is caused by environmental factors, such as ultraviolet radiation, pollution, cigarette smoking, and inadequate nutrition. During the aging process, oxidative stress is attributed to both augmented ROS production and reduced levels of enzymatic and non-enzymatic protectors. Apart from the evident appearance of structural change, throughout aging, the skin gradually loses its natural functional characteristics and regenerative potential. With aging, the skin immune system also undergoes functional senescence manifested as a reduced ability to counteract infections and augmented frequency of autoimmune and neoplastic diseases. This review proposes an update on the role of oxidative stress in the appearance of the clinical manifestation of skin aging, as well as of the molecular mechanisms that underline this natural phenomenon sometimes accelerated by external factors.

## 1. Introduction

### 1.1. Human Skin Tissue Structure and Biology

**Figure 1:**
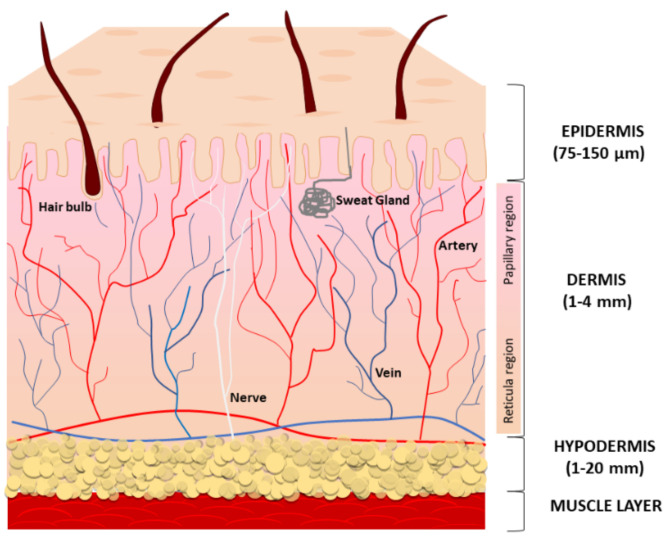
A schematic representation of the structure and functions of the skin. Human skin is composed of three layers: the epidermis (the top layer), the dermis (the middle layer), and the hypodermis (the bottom fatty layer).

The skin is the largest organ of the human body and is composed of three compartments: the epidermis, the dermis, and the deeper subcutaneous fat tissue (also referred to as hypodermis) (Figure 1).

The main function of the epidermis is to provide a protective barrier against microbes, environmental pollution, and ultraviolet (UV) radiation. The epidermis is avascular and mostly composed of multiple layers of keratinocytes at different differentiation levels. Immune cells infiltrating the epidermis, especially Langerhans cells, counteract infections. Melanocytes located at the basal layer of the epidermis produce and distribute the melanin pigment to surrounding keratinocytes, ensuring the absorption of a broad spectrum of solar irradiation wavelengths and consequent protection from UV radiation [1], whereas Merkel cells serve as a touch-perception receptor [2]. The multilayered stratum corneum (the outermost layer) consists of anucleated, densely keratinized keratinocytes and lipid-laden extracellular matrix (ECM), to prevent excess trans-epidermal water loss. Lipids consist of neutral molecules such as sterols, free fatty acids, triglycerides, highly nonpolar species, and sphingolipids [3]. Keratinocyte precursors proliferate symmetrically and asymmetrically. The asymmetric division generates two daughter cells with non-identical fates; one daughter remains a progenitor while the other commits to cell terminal differentiation. During the differentiation, keratinocytes slowly migrate towards the surface, finally becoming fully differentiated corneocyte cells that are continuously lost from the skin surface. Like other epithelial structures with an intense cell turnover, normal epidermal homeostasis firmly depends on staminal precursors, and keratinocyte aging is due to changes in stem cell numbers and functions [4]. Desquamation rates of the stratum corneum and the resultant renewal of the keratinocyte layer have been calculated at 40–56 days in middle-aged humans [5]. Thus, it has been suggested that the impact of a senescent phenotype is limited in keratinocytes. Accordingly, in the epidermis from the sun-protected areas of young and aged donors, p16INK4a-positive cells are mainly melanocytes and less frequently keratinocytes [6]. In the epidermis, melanocytes are highly differentiated cells rarely replaced during normal adult skin homeostasis and their turnover occurs only by stimulation as in wounding and exposure to UV [7]. Melanocyte precursors reside in the hair bulge and in the epidermis to pigment the hair and skin [8,9,10]. Additionally, some melanocyte precursors have been demonstrated in the dermis [11,12].

The dermis, derived from mesoderm, underlies the epidermis and includes sebaceous glands, hair follicles, nerve endings, blood, and lymphatic vessels embedded within connective tissue, a fiber-dense ECM due to the intense secretory activity of sparse fibroblasts. The dermal compartment also includes a wide repertoire of immune cells, including macrophages, dendritic cells, natural killer cells, lymphocytes, and mast cells [13]. Collagen fibers confer resistance to the tissue and elastin keeps skin flexible. In addition to structural proteins, the relative abundance and the distribution of adhesion proteins (fibronectin, lamina, fibrillin, tenascin), glycosaminoglycans (hyaluronan, heparin-sulfate, chondroitin-sulfate), and proteoglycans (versican, aggrecan, neurocan) define the biological properties of the dermis. This includes the retention and consequent availability of soluble factors, particularly growth factors and second small messengers [14,15,16]. Further, the physical properties of ECM may affect adult mesenchymal stem cell (MSC) proliferation and differentiation potential [17,18]. The specific architecture of ECM might contribute to MSCs’ fate via direct physical interaction with these cells. Moreover, the loss of stem cell properties that coincides with the spontaneous differentiation may be due to the response of stem cells to growth factors, which in turn are influenced by the microenvironment [19]. Histologically, many age-dependent changes affect the structural components of the connective tissue. Low biosynthesis, increased degradation, and the accumulation of unfunctional disorganized collagen and elastin fibers impair the tissue integrity during intrinsic aging, whereas exacerbated expression of collagen and elastin proteolytic enzymes in sun-exposed areas leads to the accumulation of partially fragmented elastin fibers, causing the typical solar elastosis of extrinsic aging [20]. The hypodermis, the bottom layer of cutaneous tissue, regulates body temperature and makes protection for blood vessels, nerves, muscles, and bones. It is a well-vascularized connective tissue prevalently composed of adipose tissue that forms a layer of variable thickness depending on its location in the body and scarce collagen fibers. Adipose tissue functions in thermal insulation and energy storage, whereas mesenchymal stem cells are key players in wound healing and re-epithelialization [21]. The depletion of stem cell reserves can compromise the ability to restore spontaneous tissue repair. Wound healing slows with age, thus older adults frequently have chronic wounds, with a significant impact on the quality of life of patients and their families [22]. Abnormal repeated requirements of tissue repair, such as in the case of disease-specific tissue dysfunction or chronic oxidative stress, might lead to premature consumption of the stem cell reservoir and consequent gain of senescent cells. The intense secretory activity of subcutaneous adipose tissue includes repair-inducing activators of fibroblasts and stem cells during wounds and very important signals to modulate hair follicle physiology, while bacteria-sensing adipocytes produce antimicrobial peptides, supporting innate immune responses in the skin [21,23]. Adipose-derived stem cells play a key role in protecting skin from oxidative damage and inflammation by the secretion of bioactive molecules and antioxidant factors [24,25].

### 1.2. Skin Antioxidant Defense System

To scavenge reactive oxygen species (ROS), cutaneous cells utilize a conspicuous apparatus of small antioxidant molecules and endogenous enzymes. Ubiquinol (coenzyme Q10) is a lipid-soluble intracellular and extracellular radical scavenger that protects mitochondria and key cutaneous proteins. CoQ10 also inhibits the expression of some metalloproteinases (MMPs), such as collagenase, preserving the collagen content of the skin [26]. Vitamin E is implicated in membrane stabilization, preventing lipid peroxidation and oxidation of unsaturated fatty acids [27,28]. In the skin, vitamin E level is strongly sensitive to UV-induced depletion [29], and levels of vitamin E also decrease with age [30], suggesting that impairment in its detoxification activity might be involved in both natural and photo-accelerated aging. Vitamin C acts by removing free radicals and repairing oxidized vitamin E [31]. Moreover, in the skin, vitamin C is implicated in procollagen synthesis and collagen cross-linking [32,33,34]. The function of superoxide dismutase (SOD) is to catalyze the breakdown of superoxide radical anion (O_2_) into hydrogen peroxide (H_2_O_2_) [35]. In mammals, three different isoforms of SOD exert non-overlapping functions. The isoform that utilizes Cu/Zn as cofactors (SOD1) localizes in the cytoplasm and the nucleus [36], the isoform that binds Mn (SOD2) localizes in mitochondria, and SOD3, which also binds Cu/Zn, has been detected mainly in the extracellular space [37]. In vivo studies in mice evidenced that all three SODs impact skin aging [38,39,40]. In animal models, SOD2 deletion corresponds to the more dramatic phenotypes with the thinner epidermis, atrophy of the dermal connective tissue, reduced complexity of the extracellular fiber network, and a smaller amount of the subcutaneous fat tissue, all of which have been described as major characteristics [39,41]. Interestingly, Velarde et al. demonstrated an age-dependent effect of SOD2 depletion in mouse skin. In old mice, SOD2 deficiency delayed wound closure and reduced epidermal thickness due to exhaustion of premature epidermal stem cells, whereas in young animals SOD2 deficiency stimulated wound closure, sustaining epidermal differentiation, despite the induction of cellular senescence in keratinocytes [42]. In human senescent skin, fibroblasts that develop a growth arrest, and morphological and functional changes, demonstrated an adaptive upregulation of the SOD2 at mRNA and protein levels due to increased ROS concentrations [43,44]. An important enzyme that detoxifies hydrogen peroxide (H_2_O_2_) is the peroxisomal localized catalase (Cat) [45]. In the aged human dermis, Cat activity is lower with consequent elevation of H_2_O_2_ concentration [43]. By contrast, a parallel increase in ROS and Cat activity has been observed in the epidermis [46]. Another supporter of the antioxidant capability of the cell is the tripeptide glutathione (GSH). The GSH acts as a scavenger because of its thiol functional group. During the reaction, GSH is oxidized by reactive oxygen radicals and makes a dimer with another GSH (GSSG). GSH can be retaken in a reducing enzymatic reaction by the glutathione reductase consuming NADPH [47]. The mouse model demonstrated that not only the absolute amount of the oxidized GSSG but also the GSSG:GSH increases in the dermis during aging [48]. In addition to its role as an antioxidant, GSH is also a cofactor for many metabolic processes. In humans, all eight glutathione peroxidases (GPXs) are known to reduce hydrogen peroxide in water and stop lipid peroxidation [45]. Overall, the endogenous antioxidant capacity (enzymatic and non-enzymatic) of the skin is lowered with age, and the aged skin is more vulnerable to external factors, especially UV radiation, pollution, and microorganisms [49]. Since the epidermis is more exposed to external stimuli than the dermis, the ROS load is higher in the epidermis compared to the dermis [50]. Correspondingly, defensive enzymes and non-enzymatic antioxidants are present in higher concentrations in the epidermis than in the dermis [51]. Particularly, small antioxidants such as vitamins C and E, glutathione, and ubiquinol, and defensive enzymes such as Cat and SODs are concentrated in deeper layers of the stratum corneum [29,52,53]. From the biological point of view, this might correspond to more accurate protection of epidermal stem cells that mostly reside at the dermal–epidermal junction. On the other hand, the production of ROS in the epidermis occurs in the deepest layers, especially at the basal layer, since in the final phase of the differentiation process, keratinocytes of the stratum corneum lose their nuclei and organelles [54]. Moreover, the promelanogenic effect of solar radiation promotes the formation of free radicals related to the melanin biosynthetic pathway at the dermal–epidermal junction [55]. Particularly, in fair-skinned individuals, pheomelanin is responsible for free radical generation in melanocytes even in absence of UV [56,57]. Likewise, carriers of melanocortin 1 receptor (MC1R) variants presenting a shift in melanin synthesis from eumelanin to pheomelanin and consequent elevation of reactive oxygen species are at increased melanoma risk, independent of their sun exposure [58].

### 1.3. A Brief Introduction to Skin Aging

**Figure 2:**
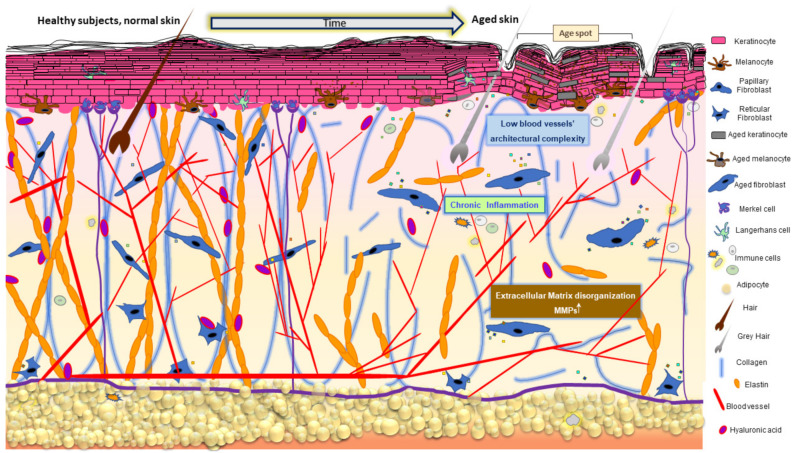
Age-related changes in the skin. Skin aging results in cumulative detrimental effects characterized by abnormal ECM organization, pigmentary changes, loss of subcutaneous fat, hair greying, minored hair density, decreased sebaceous gland function, and low-grade chronic inflammation. Cellular and molecular events reviewed in the text describe the impact of oxidative disequilibrium on these time-dependent and/or extrinsically accelerated tissue transformations.

Skin aging is a multifactorial biological process macroscopically manifested by modification of its appearance due to the progressive decline of physiological functionality (Figure 2).

Fine wrinkles, tissue atrophy with minor elasticity, and remarkable dryness often accompanied by pruritus are the most common phenotypic changes in aging observed in all skin areas [59]. However, they diversify among different anatomical regions and within diverse ethnical groups [60,61]. The subcutaneous adipose tissue is decreased in some body areas, especially the face, shins, hands, and feet, explaining the visible volume reduction, while in other body areas, peculiarly the abdomen in males and the thighs in females, it is augmented. Anatomical differences emerged by the comparative analysis of facial and abdominal adipocyte gene expression profiles, suggesting a possible implication in the diverse modification of subcutaneous tissue of these body areas during aging [62]. Sebaceous glands progressively increase in size, but their secretory output is attenuated in aged individuals [63]. There is a progressive decline in the density of hair follicles, and the hair shaft diameter is frequently smaller [64]. At the cellular level, aging is characterized by the accumulation of senescent cells in both the epidermis and the dermis and by a significant depletion of stem/progenitor cells [65]. Since MSCs do not escape the deleterious effects of natural aging, their propensity to senesce is firstly determined by intrinsic factors [66]. Aging affects MSCs from a quantitative (stem cells exhaustion) and a qualitative point of view, since advanced age subcutaneous MSCs lose their osteogenic potential and in turn augment the adipogenic potential [67]. In line with this idea, Orciani and collaborators demonstrated that MSCs isolated from the skin do not have an efficient antioxidant defense system, but their integrity is preserved by the surrounding microenvironment of the niche [68]. Increased intracellular ROS and lower SOD activity characterizes aged MSCs in both undifferentiated and differentiated conditions. Diabetic subcutaneous MSCs displayed lower proliferation activity, upregulation of pluripotent staminal markers, and a propensity for neurogenic differentiation. At the same time, normal MSCs cultured in a hyperglycemic milieu showed an ROS-dependent similar phenotype characterized by low proliferation and migration, senescent-prone phenotype, and a relatively immature state with an inclination to neuron-like differentiation [69]. This study demonstrated that metabolic dysfunction, frequently arising in the elderly, might impact skin aging and its reparative potential.

Replicative senescence is principally the result of repeated cell division that induces gradual shortening of telomeres [70]. Although cellular replication is a major contributor to telomere dysfunction, it has been largely documented that telomere attrition is accelerated when cells are exposed to mild, eventually chronic stress, leading to reduced replicative capacity and a phenotype similar to replicative senescence [71,72,73]. The physiological elimination of senescent cells is mainly modulated by the immune system, but the mechanisms involved are not yet completely elucidated. In addition, tissue degeneration may help senescent cells to escape from immune clearance [74]. In the complicated context of organismal aging, it is not known to what extent continuous proliferation contributes to regulating the number of senescent cells, and whether age-related impairment of the immune system function contributes to the accumulation of senescent cells in old individuals [75]. Furthermore, senescence in fibroblasts matches with resistance to apoptosis caused by UV radiation [76], a characteristic that could influence the persistence of these cells. In a restricted number of clinical trials, pharmacological treatments, termed “senolytics”, have been tested to remove senescent cells from the body [77,78,79]. The prevalent mechanism of action of senolytic therapies involves the induction of apoptosis in senescent cells or the stimulation of immune cells to clear senescent cells. Pharmacological clearance of senescent cells markedly enhances health span [80,81,82]. Due to the discovery of practicable “therapeutic” intervention, it has been proposed to consider aging as a real “disease” [83]. Cell-specific functional diversity impacts senescence differently [84,85]. Though one might expect cells with a rapid turnover to be senescence prone, this does not seem to be the case for keratinocytes, since keratinocytes, damaged or not, are physiologically quickly replaced by new differentiating cells. Instead, fibroblasts could progress through the senescence program and accumulate functional defects, impacting tissue integrity due to their limited proliferation rate. The fact that the senescence-associated phenotype is mostly referred to as long-living post-mitotic cells underlies that elements other than those associated with replication play a relevant role in the acquisition of the senescent phenotype [86]. A plethora of stresses can provoke premature cellular senescence, including UV radiation, mitochondria dysfunction, oxidative stress, DNA damage, epigenetic alteration, and expression of some oncogenes [87,88]. Thus far, cellular senescence is not only a time-defined process: each cell experiences senescence conforming to its proliferation speed and its history. Therefore, in real life, a tissue that underwent exclusively intrinsic aging does not exist. Since skin is the interface between the body and the external environment, it is constantly or intermittently in contact with extrinsic stimulation (e.g., ultraviolet light exposure, pollution, smoking, chemotherapy, radiotherapy, cosmetics, microbial insults, trauma) in addition to intrinsic factors (e.g., time, genetic factors, hormones, comorbidities) that can impact the senescence of its cells and on the overall decline of tissue function. Thus, it is considered the organ of choice for aging studies [89,90,91]. Moreover, due to the skin’s accessibility, it is a useful model to test translational approaches in the regenerative medicine field [92,93]. The neologism of “exposome” has been recently introduced to explain the complex exposures we face throughout our lives, and encompasses air pollution, climate factors, infections, the food we ingest, the objects we touch, and the psychological stresses [94]. Exposomes exacerbate tissue damage, accelerating the aging process [95]. UV radiation is the most potent extrinsic driver of age-related change in the skin, known as “photoaging”. Photoaging accounts for approximately 80% of facial aging [96]. Except for pigmentation, which presents the opposite feature in chronological aging (hypopigmentation) [85,97,98] and in photoaging (hyperpigmentation) [99,100], intrinsic and extrinsic skin senescence demonstrated several types of overlapping pathogenic molecular signaling. The common feature of both types of cutaneous aging is the generation of ROS, impacting DNA, protein, and lipid damage and the disorganization of the ECM [101]. During naturally occurring aging, collagen and elastic fibers are partially degraded but form a wider-mashed network. During extrinsic aging, the dermis strongly loses collagen type I (Col-I), III, and VII expression [102]. Additionally, the migration of neutrophils after inflammation or UV exposure strongly accelerates collagen and elastin degradation due to their intense production of MMPs and elastases [103]. In mice, a comparative analysis of the gene expression profile of young and old animals revealed that most of the differentially regulated genes encompassed those induced by oxidative damage and associated with energy metabolism, mitochondrial function, and turnover [104]. Furthermore, the relevance of redox equilibrium in the aging process is supported by the proof that human skin fibroblasts from progeria patients, a premature aging syndrome, show a significant reduction in SOD2, Cat, and GPX expression and activity at the basal level. Moreover, these cells also demonstrated impaired scavenger activity under chronic oxidative stress conditions [105].

At the low level, ROS are the first line of defense and are involved in various physiological functions; however, an excessive amount of ROS or an insufficient endogenous defense system can compromise intracellular redox homeostasis. A large amount of ROS activates mitogen-activated protein kinases (MAPKs) and critical transcription factors such as nuclear factor-κB (NF-κB), nuclear factor erythroid 2-like (Nrf2), and c-Jun-*N*-terminal kinase (JNK) and transcription factor activator protein 1 (AP-1) [106]. Nrf2 is one of the most important transcription factors in the cellular response to oxidative stress. Nrf2 cytoprotective action concerns mainly antioxidant enzymes such as glutathione S-transferase (GST), heme oxygenase-1 (HO-1), quinone reductase NAD(P)H (NQO1), Cat, SODs, UDP-glucuronosyltransferases (UGT), epoxide hydrolase (EPHX), γ-glutamylcysteine ligase (GCL), glutathione reductase (GR), and thioredoxin reductase (TrxR) under normal and critical circumstances [107]. For this reason, natural Nrf2 modulators received notable attention in dermatology [108]. AP-1 activation elevates the expression of MMP1, 3, and 9 in fibroblasts and keratinocytes [109,110]. AP-1 and NF-κB inhibit TGF-β, which is responsible for Col-I and connective tissue growth factor (CTGF) production [111,112]. ROS-triggered activation of NF-κB also drives an elevation of proinflammatory cytokines (IL1, IL6, and TNFα) [113], provoking localized phenotypic changes that are independent of the systemic immune system function [114,115]. Stress-induced senescence is associated with a marked pro-inflammatory secretory profile of dermal and epidermal cells [116]. Reduced ability to manage persistent inflammation contributes to the occurrence of skin inflammaging, immunosuppression, and skin cancers [117,118]. Since mitochondria are the main source of intracellular free radical production [119], dysfunctional mitochondria contribute to the aging process [120]. At the molecular level, a typical marker of aging is the low expression level of mitochondrial electron transport chain proteins [121]. A disturbance of reductive overload of the mitochondrial respiratory chain leads to uncontrolled ROS accumulation [122]. Characteristic features of the aged dermis and epidermis are the presence of damaged mitochondria, frequent mitochondrial DNA (mtDNA) deletions, elevated ROS levels, and oxidative stress [123]. Skin aging and stem cell senescence are characterized by lower mitochondrial complex I–IV activity [124]. A 4977-base-pair extended region of mtDNA, coding for genes of complexes I, IV, and V respiratory chain, is frequently deleted in the aged human skin [125]. This ″common deletion″ positively correlates with sun exposure and skin wrinkles [126]. Damaged mitochondria are selectively sequestered in double-membrane vesicles and cleared away by lysosome-dependent mitophagy (selective autophagy of mitochondria). Mitophagy is utilized under cellular stress conditions to preserve healthy mitochondria or to regulate homeostasis when an excess of mitochondria is present [127]. Older skin had a significantly fragmented mitochondrial network, indicating poor recycling and excessive mitophagy [128]. Signals of mitochondrial dysfunction are sensed by the mammalian target of rapamycin (mTOR) or AMP-dependent protein kinase (AMPK) and calmodulin [129,130]. Conditions that activate mTOR (deregulated autophagy, oxidative stress, or systemic inflammation) when the cell cycle is blocked lead the cell to a hypertrophic senescence state [131]. Further, ROS activates the PI3K/mTOR/S6K axis, leading to an increase in cell volume and protein content [132]. Thus, ROS can be linked to cell senescence, not only through damaged macromolecules but also through mTOR. Notably, the activation of mTOR, which promotes cell growth even though the cell cycle is blocked downstream, causes hypermitogenic senescence. Increased cellular function (hypertrophy, pro-inflammatory and hypersecretory phenotypes) in a context of reduced or absent proliferation resembles senescence caused by DNA damage, and it is considered a distinctive marker of hypermitogenic arrest [133]. Hypertrophy, hyperplasia, and hypermitogenic phenotype [134,135] by themselves may cause changes in skin appearance. In the mouse model, overexpression of the insulin-like growth factor II gene (IGF-2) in keratinocytes causes overgrowth of the skin, as denoted by wrinkling [136].

## 2. The Role of Oxidative Stress in Chronological Senescence of the Skin

In human skin, consistent with all other organs, physiological aging is the natural consequence of the passage of time. Skin that ages only by intrinsic factors virtually does not exist. Thus, it is generally considered that skin that exclusively undergoes intrinsic aging is usually present in body areas habitually unexposed to sunlight. Naturally, aged skin presents fine wrinkles, dryness, thinning, and augmented temperature sensitivity. During skin aging, sebocytes decrease the size and secretory activity with a significant repercussion in the surface lipid amount and skin hydration [137]. In menopausal women, a significantly higher pH of the hydro-lipid film surface and a decrease in sebum production have been observed, whereas measurements of trans-epidermal water loss showed a minimal variation in stratum corneum hydration [138]. Chronic itching is likewise common in the elderly due to dryness. However, it may be caused by the age-related alteration of touch to itch sensation due to dysfunctional Merkel cells [139]. During aging, keratinocytes acquire a typical shorter and fatter morphology, while corneocytes become bigger as a result of lower epidermal turnover [140]. In old skin, the stratum corneum is not replaced as quickly, so the skin appears rough and dry. Extreme skin dryness (xerosis) is more susceptible to irritant dermatitis [141]. As mitosis of keratinocyte precursors in the basal layer of the epidermis is slowed down, healing requires a longer time. Moreover, there is a reduction in the surface contact between the epidermis and dermis, which aggravates the delivery of oxygen and the nutrients in the epidermis [141]. The aging-associated anatomical change includes the decrease in the number and size of blood vessels, and low architectural complexity that explains not only the impaired nutrition supplementation but also the removal of metabolic debris and toxins [142]. Vascular permeability changes, especially in the superficial dermis, produce adaptative remodeling of the epidermis such as the decrease in several cell layers, thus reducing the thickness [143,144]. At the tegumentary level, dermis fibers undergo fragmentation processes and finally lysis. Elastic fiber degeneration is faster than that of collagen fibers [144]. The age-related decrease in sweat glands might be responsible for impaired metabolic waste removal and consequent accumulation of toxins on the skin [145]. With aging, hair becomes thinner on the scalp and terminal hair follicles are progressively miniaturized [146]. As can be easily observed, the natural hair color is lost due to a reduced transfer of melanin from follicular melanocytes to hair keratinocytes [146]. Intrinsic aging is mainly regulated by genetic factors affecting the entire body. Most of the genes associated with a younger appearance are directly or indirectly linked with protective factors such as DNA repair, response to oxidative stress, cell replication, protein metabolism, or ECM architecture [147]. Single nucleotide polymorphisms (SNPs) for genes responding to NAD(P)H dehydrogenase (NADPH), SOD2, SOD3, Nrf2, Cat, and GPX1 have been correlated to skin aging [147,148]. Since the activation of α-melanocytes hormone (α-MSH)-dependent intracellular signaling not only regulates melanogenesis but also cellular defense mechanisms in the dermis and epidermis, individuals carrying loss-of-function polymorphic variants of its receptor, the MC1R, present a reduced capacity to counteract oxidative stress and DNA damage [149,150,151,152,153]. Correspondingly, loss-of-signaling MC1R polymorphisms are linked to an increased risk of melanoma and other skin cancers [154,155,156]. Further, three different MC1R common polymorphisms, namely R151C, R142H, and D84E, demonstrated a significant correlation with photoaging [157]. In dermal fibroblasts, α-MSH modulates collagen metabolism by improving the orientation of the collagen fibers [158,159,160]. α-MSH may drive the healing into a more regenerative/less scarring pathway by counteraction the pro-fibrotic action of TGFβ [160,161]. Recently, a higher prevalence of coarse collagen, pixel, and vessel density in photo-exposed areas has been associated with MC1R polymorphic variants [162]. Apart from the genetic difference, in the epidermis, the production of proopiomelanocortin (POMC), the precursor of α-MSH, increases with age, whereas its receptor decreases, indicating that α-MSH-dependent intracellular signaling is deeply involved in the skin aging process [163]. Other genes involved in skin color regulation, including heterogeneous nuclear ribonucleoprotein (*hnRNP*), agouti signaling protein (*RALY*/*ASIP*), basonuclin 2 (*BNC2*), and interferon regulatory factor 4 (*IRF4*) have been associated with pigment spot and generally the aged appearance of the skin through pathways independent of the pigment production [164]. Regarding the dermis, SNPs of collagen type 1 alpha-2 gene (*COL1A2*), *COL17A1*, *MMP3*, *MMP9*, and *MMP16* are linked with features of aging [147,148,165,166]. Further, genetic variants of TNF receptor superfamily member 6b (*TNFRSF6B*), *TNFRSF8*, *IL6*, and *NOS1*, involved in inflammation, have been correlated with wrinkle risk [147,148,166]. Altogether, genetic data confirm the involvement of oxidative stress and inflammation in aging propensity. Epigenetic changes also participate in the regulation of the homeostasis and regeneration of aged skin [167]. Skin samples from the elderly display increased heterogeneity of global methylation patterns that are characterized by reduced connectivity of gene expression networks probably mediated by methylation-dependent changes in transcription factor binding [168]. On the opposite, studies restricted to the epidermis showed very similar methylation patterns in the epidermis of young and old individuals, demonstrating a limited destabilization of the epigenome of this tissue compartment during aging [169]. Slowing the cell cycle coincides with the lengthening epidermal turnover rate resulting in less effectiveness in wound healing and desquamation in older adults [170]. Physiological changes in skin color due to aging alone are minimal. With the aging of the non-sun-exposed skin, pigment is light and homogenously distributed. An inverse relationship between age and the proliferative activity of melanocytes has been observed independently of skin phototype [97,98,171,172]. Thus, reduced skin pigmentation and tanning response after UV exposure make aged skin more vulnerable to solar radiation [173,174]. When occurring, the white spots in aged skin are usually stellate pseudo scars or idiopathic guttate hypomelanosis [173]. Recently, Victorelli and collaborators proposed a central role of senescent melanocytes in skin aging priming because their secretory activity diminishes basal keratinocyte proliferation and guides the epidermis to atrophy in in vitro 3D human epidermal equivalents [175]. This seems to be due to paracrine CXCR3-dependent mitochondrial ROS activation, which in turn induces telomere dysfunction in neighboring cells. Epidemiologic studies further evidenced age-related changes in melanocytic nevi [176]. During life, there is a gradual lessening in the number of common and atypical nevi [173,177]. In the skin, cells positive for senescence markers p16INK4a and senescence-associated lysosomal beta-galactosidase (SA-βgal) physiologically accumulate with age in the epidermis and the dermis [178,179,180]. Senescent cells are in a non-proliferative metabolically active state and constantly produce several pro-inflammatory mediators, proteases, and mitogenic factors in a state known as the “senescence-associated secretory phenotype” (SASP) [181]. SASP spreads senescence in neighboring cells, tissues, and organs, as it functions in an autocrine and paracrine manner [182,183]. Studying human in vitro skin equivalent, Adamus and collaborators demonstrated that the age of the keratinocyte’s donor strongly impacts the model’s quality [184]. For these cells, non-univocal results have been reported in the literature regarding the expression of the proliferation marker Ki-67 in skin biopsies since inverse correlation with p16INK4a was observed by some authors [184,185], whereas others reported no change [179] or an increase [186]. From a functional point of view, biological aging manifests as a decreased physiological reserve that oxidizes DNA, enzymes, and proteins. Structural and functional dermal changes are the major contributor to the skin aging appearance [187,188]. The rate of proliferation and the total number of fibroblasts, the most abundant cell type of the dermis, decreases progressively with age, suggesting that lower fibroblast cellularity could be responsible for the occurrence of age-related features, particularly for the decreased collagen production that has been estimated at 75% lower in people aged ≥80 years [180,189,190]. Studies at a proteomic level highlighted age-dependent dermal fibroblast secretion patterns including inflammatory regulators, mitogens, angiogenic factors, and MMPs. MMPs degrade matrix components, causing less effective epidermal anchorage, skin relaxation, and decreased interstitial fluid [191,192]. More in detail, ‘‘skin-aging-associated secreted proteins”, or SAASP, in addition to the classical SASP, are enriched in molecules involved in elastic fiber formation, glycosphingolipids, and sphingolipid metabolism. Apart from ECM organization, SAASP has been associated with the regulation of insulin-like growth factor (IGF) transport and uptake by IGF binding proteins (IGFBPs), alteration in adherent junctions’ interactions (N-cadherin and Cadherin-11), glucose and carbohydrate metabolism, and capability of glutathione synthesis and recycling. Specifically, a lower level of expression of glutathione-s-transferase (GST) in middle-aged and old fibroblasts explains the of loss detoxification capability in intrinsically aged skin [193]. Naturally, aged dermal fibroblasts with reduced mechanical force downregulate TGFβRII, thus impairing the TGFβ/SMAD signal transduction pathway [194]. Reduced contractile force and migratory potential of old fibroblasts have been proposed as biomarkers of dermal aging processes [195,196]. Abnormal TGFβ/SMAD3 signaling in turn results in repression of CTGF-dependent type I collagen synthesis as well as stimulation of MMP1-induced type I collagen degradation, leading to dermal thinning [112,197]. Reciprocal regulation of TGFβ and ROS strongly impact normal and pathological connective tissue remodeling [198,199]. Reduced levels of tissue inhibitors of metalloproteinases (TIMPs) are a consequence of natural aging [200]. Interestingly, Salzer and collaborators demonstrated that aged upper (papillary) dermal fibroblasts progressively acquire the characteristics of the lower (reticular) dermis, with a minor expression of ECM proteins and gain of adipogenic markers [201]. Since it has been proposed that reticular fibroblasts are in a more advanced stage of differentiation than papillary fibroblasts, it seems plausible that the transition from the papillar to reticular state is related to the relentless terminal differentiation process of post-mitotic cells [202,203]. Another recent study proposed a specific feature of chronological age-related partial loss of dermal fibroblasts described as “loss of cellular identity” [204]. In detail, old papillary fibroblasts presented fewer papillary and more reticular gene expression profiles, while the reticular counterpart presented a less decided reticular gene expression profile [205]. This observation is of interest since dermal ECM produced by papillary fibroblasts better supports epidermal longevity compared to reticular-generated ECM [206]. A comparative analysis of matched papillary and reticular fibroblasts revealed that intrinsic aging differentially impacts these two cell populations. Colony growth at low culture density and growth rate in mass is strongly reduced in papillary fibroblasts compared to reticular fibroblasts [203]. This could be explained by the more pronounced exposition of superficial dermis to external stimuli and the consequent impact on the redox equilibrium of the papillary dermis. A recent work pointed out the attention to the effect of intrinsic age on the differentiation capacity of a restricted population of fibroblasts localized within the conjunctival junctions that connect the dermis to the hypodermis, i.e., dermo–hypodermal junction fibroblasts [207]. These cells displayed distinct age-related features when compared to papillary and reticular fibroblasts, presenting attenuated osteogenic differentiation potential, no adipocyte differentiation capacity, and unmodified chondrocyte differentiation capacity [207]. Intrinsic aging also reduces subcutaneous fat with accompanying increased vessel disorganization, loss of cellularity, and vascularity [208]. The lack of microvascular organization in the skin has retained the cause of age-related deficits in the diffusive transport capacity of the skin vasculature [142]. During chronological aging, the body undergoes a continuous increase in systemic low-grade inflammation, a process known as ″inflammaging″ [209,210]. Such chronic aging-related inflammatory response is marked by an increase in systemic levels of IL1, IL6, and IL33, as well as TNF, IFNγ, and GM-CSF [211,212]. The relationship between the senescence of skin-resident immune cells and inflammaging has not been fully understood yet. In aged skin, decreased self-renewal capacity of in situ Langerhans cell progenitors causes a reduced number of their mature counterparts. Additionally, the lower migration propensity of these cells in geriatric individuals contributes to impaired skin barrier integrity [212,213]. In addition, Langerhans cells in aged skin express a low amount of human beta-defensin-3, which is an important antimicrobial peptide produced in response to microbial infection or skin dysbiosis [214]. Thus far, the lessening of skin barrier function facilitates pathogens and pollution invasion driving low-grade chronic inflammation. Thus, when not locally restricted, the presence of inflammatory markers in senile skin might mirror a more complicated dysfunction of the immune system. Inflammaging has a widely recognized role in most common age-related diseases such as Alzheimer’s Disease, Parkinson’s Disease, heart diseases, multiple sclerosis, atherosclerosis, cancer, type II diabetes, and many others [215,216]. Finally, a defective epidermal barrier resulting from aged skin is also linked to age-related alternations to the gut and skin microbiome consisting of millions of bacteria, fungi, and viruses [217,218]. Investigations regarding the skin microbiome and its association with common clinical skin aging parameters including pigmentation, wrinkles, and texture demonstrated a clear age-dependent microbial signature attributing a causative effect of the altered skin microbiome in skin aging promotion [219].

## 3. The Role of Oxidative Stress in Extrinsic Aging of the Skin

In addition to the physiological mechanism of aging, exposure to UV light and environmental pollutants accelerate the acquisition of the aged phenotype. UV light is the major extrinsic agent responsible for skin aging. Premature photoaged skin typically presents with increased thickness of the epidermis, irregular pigmentation, and dermal connective tissue damage including the typical solar elastosis, laxity, dullness, roughness, and alteration of the vascular system [220]. The primary modification of the dermis during photo-accelerated aging regards structural components: collagen, elastin, and glycosaminoglycans. In older skin, the collagen network looks disorganized, and the ratio of collagen type III (Col-III) to I has been shown to increase due to less Col-I production [221]. Dissimilar to chronologic skin aging, MMP-induced collagen degradation and elastin degeneration are key mechanisms in photoaging [222]. In addition, the reduction in fibrillin structures and Col-VII involved in the bond between epidermis and dermis contribute to wrinkling formation [223]. Photoaging occurs principally due to UVA and UVB irradiation, which based on their distinct physical properties, induces different partially overlapping biological responses including abnormal ROS accumulation and/or DNA damage in both the epidermal and dermal compartment [224]. The fact that photoaging is largely due to oxidative disequilibrium is confirmed by the efficacy of topical antioxidants in UV-induced damage prevention [53,225]. Overall, depending on the cell type and the intensity of the stress, cells can either transiently block the cell cycle and repair the damage before restarting cell proliferation or enter apoptosis if the sensed damage is too serious. Therefore, sub-cytotoxic acute or chronic stress can induce premature senescence. Several pieces of evidence revealed that under the same conditions, human fibroblasts predominantly respond via senescence, while epithelial cells prefer to exert apoptosis [185,226]. Together with the cell-specific turnover rate, this partially explains the preferential detection of the senescent markers in the dermal compartment compared to the epidermal one. The magnitude of photodamage depends on constitutional factors, e.g., skin phototype (skin color, capacity to tan) and frequency and/or intensity of sunlight exposure [227]. The main cause of sunburn is UVB radiation. Skin UVB exposure results in the enhancement of NF-kB signaling. This signaling pathway is responsible for the augmented secretion of inflammatory mediators (IL1, IL3, IL6, IL8, IL7, IL10, and TNFα) by keratinocytes [228]. Exposure to UV light also activates lipoxygenase and cyclooxygenase pathways (LOX and COX-2), resulting in the production of leukotrienes and prostaglandins [228,229]. Prostaglandin E-2 (PGE-2) has a prominent role in the development of skin damage associated with both intrinsic and extrinsic aging. Studies focused on full-thickness sun-protected skin showed that both PGES-1 (PGE Synthase-1) and COX-2 expression progressively increased in dermal fibroblasts with age [230]. Additional studies confirmed higher COX-2 enzyme expression in chronologically aged skin and photoaged skin compared to young skin [231]. In addition, inflammatory cells such as lymphocytes, eosinophils, mast cells, and mononuclear cells are incremented [232]. This complex cascade of inflammatory events might determine local and potentially also systemic immunosuppression, which may not only undermine the control of dysplastic and neoplastic skin lesions but also favor immuno-pathological and infectious skin diseases [233]. Lower energy UVA rays penetrate deeply to the dermis, generating ROS that exacerbate the UVB-dependent mutagenic risk [234,235]. If not repaired, DNA mutations persist through subsequent cell subdivisions causing precancerous lesions (actinic keratosis) and skin cancers (basal cell carcinomas BCC, squamous cell carcinomas SCC, and melanoma).

UV radiation stimulates keratinocytes and fibroblasts to secrete numerous cytokines that are responsible for the proliferation of melanocytes and transient melanogenesis activation [236,237,238,239]. Fibroblasts isolated from habitually sun-exposed skin produce a greater amount of promelanogenic factors, such as hepatocyte growth factor (HGF), stem cell factor (SCF), and keratinocyte growth factor (KGF), whereas keratinocytes secrete α-MSH, SCF, prostaglandins, endothelin (ET), KGF, granulocyte-macrophage colony-stimulating factor (GM-CSF), and basic fibroblast growth factor (bFGF) [239,240,241]. The pigmentation response is an adaptation that prevents further DNA damage. Consequently, photoaged skin is characterized by irregular areas of pigmentation and hyperpigmented lesions [242,243]. Clinically, these dyspigmentations are known as solar lentigines (SL). Hyperpigmentation of this nature occurs due to changes in melanin synthesis, distribution, and turnover maintained by the focally increased number of melanocytes in the epidermis [244]. However, since decreased melanin removal rather than increased melanin production is considered the cause of hyperpigmented age spots, keratinocytes play a central role in this type of hyperpigmentation [245]. Melanin-loaded keratinocytes in SL show a lower proliferative capacity, altered differentiation, and are resistant to apoptosis. In addition, enhanced expression of p16INK4a and enlarged cell body size in SL suggest that keratinocytes are in a senescent condition [245]. In line with the idea that intrinsic factors could make the difference in the impact of extrinsic insult responses, darker skin types produce fewer radicals in the UV light compared to light skin types, whereas no differences were observed in the visible and infrared light [117]. This is mostly due to the prominent presence of pheomelanin in lighter skin amplifying UV-induced ROS formation [246]. Thus, in the dermatology practice, skin-type-specific sun protection is desired [247]. Apart from UV light, other radiation sources significantly impact skin aging. Infrared radiation, visible light, and artificial light promote ROS formation [248]. Infrared radiation causes photoaging and erythema, whereas visible light and artificial light stimulate inflammatory factors and hyperpigmentation [248]. Extensive clinical data demonstrated that outdoor (smog, ozone, particulate matter, etc.) and indoor (tobacco, solid fuel) pollution act in synergy with UV light in premature skin aging appearance [249,250]. A correlation between the number of years and packs of cigarettes smoked and the degree of skin aging has been documented [251]. Smokers have less skin elasticity and a reduced amount of collagen due to the low level of collagen synthesis [249]. Smoking promotes keratinocyte dysplasia and roughness of the cutis, and a dose-dependent relationship between wrinkling and smoking has been demonstrated [89,252]. In vitro exposure to tobacco extract induced the MMP1 expression by activating the aryl hydrocarbon receptor signaling pathway in human keratinocytes and fibroblasts [253]. An in vivo study confirmed a higher level of MMP1 expression in the dermis of smokers compared to non-smokers, resulting in collagen and elastin breakdown [254]. Due to an increase in ROS, reactive nitrogen species (RNS), DNA damage, protein, lipid oxidation, and altered mitochondria homeostasis, pesticides are a potential risk for skin aging and carcinogenesis [255,256]. Among exposome factors, dicarbonyl compounds such as glyoxal and methylglyoxal displayed the capacity to trigger oxidative stress-induced senescence in fibroblasts and keratinocytes [257,258,259]. These dicarbonyl compounds come from environmental exposure or food consumption [260]. However, age and metabolic diseases, such as diabetes, compromise the detoxifying activity of glyoxalases, leading to the accumulation of toxic glycation end products [261]. Accordingly, antioxidant exogenous supplementation with dietary antioxidants and/or cutaneous treatment with antioxidant molecules have been proven beneficial to improve photoaging [262]. However, the penetration in the stratum corneum of some antioxidants might be challenging. Several anti-aging strategies attempt to reduce (or avoid) exposure to UV and to reinforce the antioxidant capacity of the skin. The combination of both strategies is desired, since sun protection does not offer an appropriate protection to resist UVA-induced ROS. Numerous lines of evidence support the hypothesis that antioxidant compounds such as ascorbic acid, polyphenols, tocopherols, and other natural substances limit the concentration of free radicals, attenuating oxidative stress and slowing down the process of aging [263]. Ascorbic acid (vit C) directly scavenges ROS generated by UV radiation and promotes the biosynthesis of elastin and collagens [264]. In addition, phytochemicals such as resveratrol, quercetin, and green tea extract act as antioxidants to scavenge free radicals or ROS and have been reported to be effective in decreasing or retarding the progression of the aging process [265]. In addition, some phytochemicals act as anti-inflammatory agents by inhibiting the production of inflammatory mediators and cytokines and as stimulators of fibroblasts [266]. More recently, oral administration of bioactive collagen peptides to prevent skin aging has attracted more and more attention. Different formulations demonstrated efficacy in increasing skin hydration, elastin, pro-collagen I, and fibrillin, reducing wrinkle width [267,268] and increasing dermal matrix synthesis. Similarly, in preclinical animal models, oral supplementation plant polysaccharides extracted from *Tremella fuciformis* and *Sargassum fusiforme* enhanced SOD, Cat, and GPX activity significantly and decreased ROS and malondialdehyde levels, improving skin damage following UV exposure [264,269]

## 4. ROS and Hair Greying

**Figure 3:**
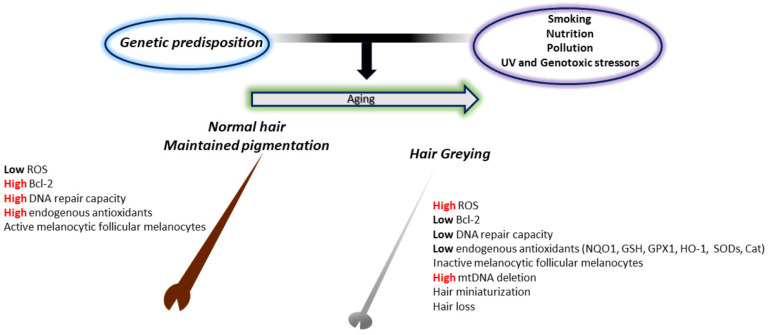
The involvement of oxidative stress in the greying process. In young and healthy subjects, melanocytes in pigmented hair follicles can deal with the low endogenous oxidative stress caused by the melanin biosynthetic pathway. This is due to the adequate presence of endogenous antioxidant levels and the high DNA repair activity. In old subjects, however, reduced production of endogenous antioxidants and repair enzymes induce deleterious oxidative stress damage in pigment-producing cells. This results in the accumulation of senescent inactive melanocytes around the dermal papilla and at the outer root sheath, no melanocyte stem cells at the bulge area, and consequent a non-pigmented hair shaft.

One of the simplest indicators of aging in humans is hair greying or ″canities″. This fascinating phenomenon, characterized by loss of pigment production and deposition within the hair shafts [270], has attracted the attention of researchers for years and has led to a better comprehension of the intricate relationship between genetic, metabolic, neuroendocrine, and oxidative factors in its manifestation. Hair greying occurs in all individuals independent of sun exposure and results from the progressive loss of hair follicular melanocytes with age. This could result from numerous impaired processes, including follicular melanocyte death, migration or differentiation failure of melanocyte stem cells, or melanocyte stem cell depletion or death. Of course, when speaking about aging, the “free radical” theory is one of the most widely experimented with (Figure 3).

Excluding UV light, hair follicles (HFs) can be exposed to other sources of ROS such as intrinsic metabolic by-products [271] and extrinsic agents (inflammatory process, smoke, drugs, poor nutrition). Indeed, the frequent 4977 bp mitochondrial DNA deletion (a marker of oxidative stress) is more frequent in greying HFs than in matched pigmented follicles, and melanocyte death by oxidative stress is increased in greying follicles [272]. The presence of highly vacuolated melanocytes within the HF, a cellular appearance that is ROS induced, corroborates the ″free radical theory of hair greying″. Some grey hair melanosomes were identified within auto-phagolysosomes, suggesting the removal of damaged melanosomes [273]. To cope with oxidative stress, the HF possesses an extremely elaborate antioxidant system [274]. A progressive failing of this system has been described in studies confronting prematurely grey hair follicles to pigmented ones, evidencing a downregulation of Cat, GPX1, and SODs in the former. These are accompanied by a 20-fold reduced expression of genes involved in melanogenesis such as Tyrosinase (*TYR*), Tyrosinase-Related Protein-1 (*TYRP1*), Microphthalmia Transcription Factor (*MITF*), Paired Box-3 (*PAX3*), *POMC*, *KIT* Proto-Oncogene, Receptor Tyrosine Kinase (*KIT*), and SRY-Box Transcription Factor 10 (*SOX10*) [275]. In a study of aging, Kauser et al. have also shown that follicular melanocytes age more than their epidermal counterparts, highlighting lower expression of Cat in follicular melanocytes [276]. In the same study, TRP-2 was significantly lowered in the older epidermal melanocytes, but at the same time, it was strongly elevated in the aged follicular melanocytes [276]. Compellingly, TRP-2 depletion has not been confirmed in eyelashes and eyebrows of the same patient group, suggesting possible anatomical differences between hair follicles of these locations. Lower antioxidant capacity due to depletion of TRP-2 from pigmented bulbar melanocytes suggests an additional antioxidant pathway that could affect hair greying. The intrinsic Cat deficiency could justify the high concentration of H_2_O_2_, which has been described in greying HFs in several reports [272,277]. Of note, the inadequate activity of Cat activity has been depicted in vitiligo, a common depigmentation disorder characterized by local or diffused destruction of melanocytes in the skin [278,279,280,281] sometimes presenting loss of hair pigmentation during disease progression [282]. Furthermore, Wood et al. have reported the inhibition of Tyrosinase, the key enzyme of the melanin biosynthetic pathway, by elevated levels of H_2_O_2_ [283]. This observation, coupled with the intrinsic ROS generation characteristic of melanogenesis [284], could justify an age-dependent melanin synthesis defect in the HF and, thus, hair greying. Accordingly, amelanotic melanocytes at the outer root sheath are somewhat less affected by oxidative damage and survive for a long time even within the white, aging hair follicles [285]. Another hypothesis has considered a possible “bleaching” phenomenon of the melanin pigment induced by H_2_O_2_ [286], even though bleached melanosomes have not yet been reported in hair follicles [287]. Of course, an obvious source of ROS is UV radiation, and Lu et al. have shown premature HF melanogenesis termination after UVB irradiation [288]. Altogether, these observations documented a high susceptibility to oxidative stress of HF melanocytes, implying an important role in the hair greying phenomenon. Ex vivo cultured human hair follicles demonstrated ROS-induced hair growth retardation, suggesting that oxidative disequilibrium intersects with diverse aspects of HF biology [289]. Oxidative stress and the aging process of HF are associated with reduced expression of the protective factor Bcl-2 [271]. Bcl-2 has a critical role in melanocyte maintenance, with knockout mice displaying accelerated greying [290]. By increasing Bcl-2 expression, melanocyte populations in the hair bulb and melanocyte stem cells in the bulge could be maintained, preventing hair greying. Finally, the compromised redox equilibrium has also been linked to the pathogenesis of androgenetic alopecia, a common heritable, androgen, and age-dependent process that results in large reductions in scalp hair density [291,292]. However, it is important to consider that ROS has an ambivalent function in hair biology. One key example of ROS relevance in HF physiology is the mitochondrial ROS-dependent activation of β-catenin and Notch signaling during HF development [293]. Based on this, photobiomodulation therapy, effective treatment for hair loss, benefits ROS-dependent stimulation of the Akt/GSK3β/β-catenin signaling pathway to drive quiescent hair follicle stem cells and alleviate HF atrophy [294].

## 5. Contribution of ROS in Common Age-Related Skin Diseases

### 5.1. Evidence of the Impact of ROS and Age-Related Skin Alteration on Tissue Vulnerability

**Figure 4:**
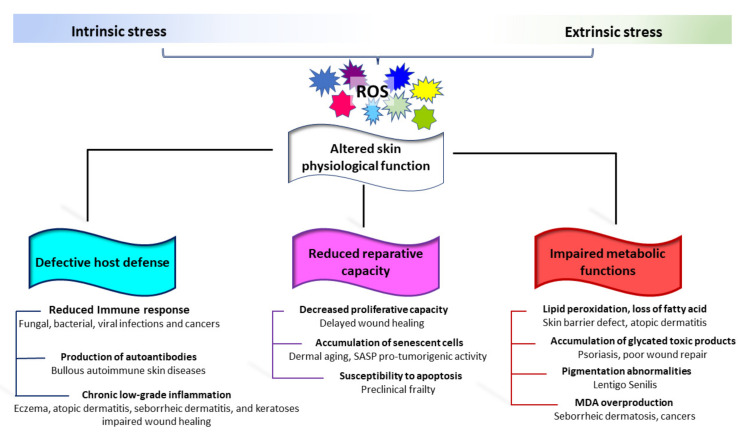
Schematic representation of the involvement of oxidative stress in common age-related skin diseases. Progressive structural and functional degeneration of the skin leaves it prone to a wide variety of very common cutaneous diseases. The onset of most of these clinical conditions involves extrinsic activation of cutaneous immune cells in a redox-dependent manner. This includes a reduced immune response, production of autoantibodies, and chronic low-grade inflammation. Structural, biochemical changes, accumulation of senescent cells, and age-related stem cell depletion impact the cutaneous repair capacity. Metabolic alterations in aged skin do not fully support the physiological function of the skin and its appendages.

Skin aging also represents a health risk, resulting in skin fragility. Age-dependent changes affecting both the adaptative and innate immune response have been well documented in older humans [295]. The immunosenescence consists of three main events: reduced immune response; increased production of autoantibodies; and inflammation (chronic, sterile, low-grade inflammation). The decrease in cutaneous immune functions facilitates a series of bacterial infections including cellulitis (particularly of the lower legs), erysipelas, necrotizing fasciitis, impetigo, folliculitis, and furunculosis [296,297]. Fungal and viral infections are also more frequent in the elderly [296,298]. Opposite, chronic, low-grade inflammation typical of elderly individuals could serve as a stimulus for the onset of autoimmunity [299]. Moreover, the possible release of self-antigens by damaged keratinocytes that contribute to the production of antibodies against neo-antigens might be enhanced by skin barrier impairment [297]. Accordingly, Bullous Pemphigoid, a subepidermal autoimmune blistering disease, is common in elderly patients [300,301]. Low antioxidant capacity/redox disequilibrium has been proven in Bullous Pemphigoid as well as in other non-age-related autoimmune skin blistering diseases [302]. Sporadically, epidermal injury following UVA and psoralen UVA (PUVA) therapies has been reported as a causative factor in attraction of autoantibodies [303,304]. Benign mucous membrane pemphigoid, paraneoplastic pemphigoid, and pemphigus Vulgaris are also more prevalent in the elderly [305]. Moreover, evidence of oxidative-stress-mediated pathogenic mechanisms has been reported for pemphigus Vulgaris [306,307,308] (Figure 4).

Structural and functional degeneration of the skin leaves it prone to various common cutaneous diseases, including eczema [309], contact and allergic dermatitis [310], seborrheic dermatitis [305], seborrheic keratoses (SK, a benign epithelial skin tumor) [311], and various forms of neoplasms. The onset of most of these clinical conditions is triggered by extrinsic activation of cutaneous immune cells in a redox-dependent manner [312,313,314]. Several age-associated characteristics of the cutaneous tissue might represent a hostile environment for wound healing. These include an abundance of ROS, persistent inflammation, and increased destruction of ECM components [315]. Further, age-associated deficits in microvascular function and poor staminal reservoir contribute to deficits in tissue healing in the older population [316]. Changes in the skin that occur in the elderly, especially dermal vascular changes, pH, and skin thickness, might directly or indirectly affect percutaneous penetration of drugs with a relevant consequence in pharmacotherapy. Albeit not linked to the classical idea of aging, several dermatological disorders are associated with the gain of senescent cells. For example, senescence-related acquired pathological pigmentary alteration including vitiligo and melasma have been described to be associated with premature senescence of the entire skin [241,317,318,319]. Harmed mitochondrial electron transport chain complex I activity in vitiligo cells, a high level of mitochondrial malate dehydrogenase activity, lower ATP production, and a diminished capacity to cope with stressful stimuli indicate an important role of mitochondrial defective functionality in the pathogenesis of vitiligo [320,321]. In vitiligo melanocytes, chronic oxidative stress drives the acquisition of a pro-inflammatory premature senescent phenotype [281]. Similarly, melasma (melanotic hypermelanosis) is a chronic relapsing hyperpigmentary disease presenting evidence of oxidative stress, subclinical inflammation, and several senescence markers [322,323]. In melasma, senescent-associated markers have been documented exclusively in disease-involved skin areas, whereas in vitiligo patients, the presence of the senescence feature has been delineated as a diffuse trait of the epidermis and the dermis in both lesional and non-lesional skin [238,281,324], suggesting an intrinsic susceptibility to premature senescence of vitiligo patients. Accordingly, for vitiligo, disease risk has been partially attributed to polygenic variants [325].

### 5.2. Contribution of ROS in Age-Related Skin Cancers

In old skin, most serious conditions are related to forms of neoplasms, such as a precancerous lesion (including actinic keratosis (AK) and lentigo maligna) resulting from the focal proliferation of mutated cells, and tumors. Merkel cell carcinoma [326], non-melanoma skin cancers [327,328], and malignant melanoma [328,329] are more frequent in the elderly due to somatic mutations accumulated over time. In melanoma, age is also an important prognostic factor. Accordingly, multivariate analyses demonstrate that age and Breslow thickness are the strongest independent adverse prognostic factors [329,330]. Both melanoma and non-melanoma occur prevalently on sun-exposed areas of the skin and present a severe response to solar radiation. UV-induced skin carcinogenesis is a complex and continuous biological process caused by different wavelengths. The two most abundant UV-induced DNA lesions, cyclobutane pyrimidine dimer (CPD) and the pyrimidone photoproduct (6-4PP), are due to UVB [331]. The increment of ROS that in turn could damage DNA by the formation of 7,8-dihydro-8-oxyguanine (8-oxodG) is largely dependent on UVA [332]. It is widely documented that sunscreens are also an important aspect of photoprotection, confirmed by their efficacy in reducing photocarcinogenesis and photoaging [333,334]. Further, several topically or systemically administered compounds confer protection against oxidative stress in dermal and epidermal cells. Vitamin D and related metabolites have been found to facilitate keratinocyte survival after UVB exposure [335], enhancing DNA repair [336,337]. Nicotinamide (NAM), a water-soluble vitamin B3 derivate, enhances DNA repair and mitigates the UV-induced suppression of immunity at the cellular level, whereas in clinical trials NAM in both topical and oral forms decreased trans-epidermal water loss and the development of cancers [338]. Thompson et al. demonstrated that nicotinamide lowered levels of 8-oxo-7,8-dihydro-2′-deoxyguanosine and cyclobutane pyrimidine dimers (two products of photolesions) in keratinocytes after exposure to both types of UV rays [339].

Independent of sun exposition, during the aging process, the skin gradually loses the capacity to counteract redox disequilibrium. Importantly, an age-related decline in the expression and activity of DNA repair proteins has been demonstrated, indicating that an organism is naturally predisposed to accumulate genomic and mitochondrial DNA mutations over time, which could further increase exposure to other ROS sources, impacting cancer probability [340]. Yet, independent of UV exposure (photoaging), intrinsic aging is perhaps the most important cancer risk factor. Furthermore, the production of ROS is a common cellular event occurring during the exposure to many, if not all, modifiable cancer risk factors, and it is generally accepted that increased ROS levels have a tumor-promoting effect in the early stages of the tumorigenic process [341,342]. Thus, mitigation of oxidative skin damage by topical or oral delivery of extrinsic antioxidant supplements may have therapeutic benefits in preventing melanoma and non-melanoma skin cancers.

Deregulated ROS in cancer are involved in cell cycle progression and proliferation, survival, apoptosis, intracellular adhesion, and cell migration. Nevertheless, from the tumor-centric point of view, senescence is a potent barrier to tumor progression. Melanocytic nevi are considered an excellent in vivo example of senescence protection of precancerous cells. Melanocytes in the nevi display features of oncogene-induced senescence, including intact telomeres, high p16INK4a expression, and SA-β-galactosidase activity [343,344,345,346,347]. This phenotype is mostly referred to as the mutational activation of BRAF and/or N-RAS, present in up to 81% of melanocytic nevi [348]. Apart from its function in cell cycle regulation, p16INK4a is involved in the prevention of ROS accumulation [349,350]. p16INK4a regulates mitochondrial biogenesis, dynamics, and function [349,350]. The central role of p16INK4a in melanocyte biology is demonstrated by the fact that individuals with familial *CDKN2A* gene (encoding p16INK4a and p14ARF) deficiency (also known as Leiden syndrome or Familial Atypical Multiple Mole Melanoma syndrome) have a characteristic accumulation of nevi and an increased melanoma risk [351]. Nevi remain growth arrested for a long time and infrequently develop into melanomas [352,353]. Thus, primary melanomas are considered a paradigm of senescence evasion. A decrease or loss of the tensin homolog (PTEN) and consequent activation of the PI3K/AKT signaling pathway may abolish senescence status, facilitating the melanocytic nevi progression into dysplastic nevi or melanoma [354]. A similar situation has been described for the keratinocyte lineage. Sasaki et al. showed ROS-induced senescence in normal epidermal keratinocytes might be triggered by upregulation of p16INK4a through demethylation in its promoter region. Since this genomic regulation has been not found in SCC cell lines, a key role in counteracting malignant transformation of normal human epidermal keratinocytes has been proposed [355]. However, the presence of p16INK4a-positive senescent cells in both benign (SK) and potentially malignant age-related skin lesions (AK) underlines the dual nature of senescence in cancer [356]. In keratinocytes, due to the renewal process regulated mainly by the proliferation/apoptosis ratio, reduced death of damaged cells causes cancer onset. At the molecular level, in keratinocyte carcinomas, p53 is the critical regulator of cell fate. P53 plays a critical role in supporting the DNA repair process and restoring genome stability. In genetically unstable cells, p53 can induce apoptosis, maintaining tissue homeostasis and tumor suppression [357]. However, loss-of-function of tumor suppressor p53 mutation, which is very frequent in the skin due to the repeated UV insult, impairs the physiological DNA repair machinery activity leading to the accumulation of further oncogenic mutations and expansion of pre-neoplastic clones [358]. Consistent with the early involvement of p53 in skin carcinogenesis, a wide number of studies documented p53 mutation in AK [359]. Interestingly, mutations at particular p53 codons are present in sun-exposed normal human skin at different frequencies depending on genetic background and lifestyle [360,361] and are considered a valid predictor of risk for basal cell carcinoma [362].

In the contest of skin cancer, pre-existing ECM alterations occurring in both aging and photoaging acquire a prominent interest in disease onset, progression, and invasion. The accumulation of senescent fibroblasts in habitually sun-exposed skin might direct stroma modification onto a tumor-promoting one [363,364]. Meanwhile, several reports have consistently suggested a mechanistic link between the aging microenvironment and neoplastic disease progress in several tissues, including the skin [365]. Aging-related fibrosis and the resulting increase in tissue stiffness have also been suggested to fuel carcinogenesis [366,367], possibly via alterations in the mechanical force balance between ECM, cell, and cytoskeleton [368]. The microenvironment surrounding benign nevi and melanomas displays greater stiffness than healthy skin, suggesting that physical assets of the tissue impact melanoma onset or progression [369]. Instead, in the contest of melanoma, modification of dermal ECM architecture and compromission of basement membrane integrity can facilitate the invasion of tumor cells. Accordingly, copious production of MMPs has been frequently reported in melanoma lesions [370]. Several studies have investigated the expression and activity of antioxidant enzymes in skin cancer cell lines, demonstrating a disequilibrium of the redox state within these cells [371]. Sander et al. reported that in human melanoma and non-melanoma skin cancer the natural redox balance is perturbed, leading to the accumulation of lipid peroxides and the formation of α, β-unsaturated aldehydes, including malondialdehyde (MDA) [372], which was shown to be mutagenic and carcinogenic [373]. MDA can combine with free amino groups of proteins, resulting in MDA-modified protein adducts, which in turn can be used as a measure for oxidative-stress-induced lipid peroxidation [374]. Intriguingly, significantly elevated MDA levels were found in SCC [375]. Primary and metastatic melanoma has a better antioxidant status than other skin tumors, including non-melanoma skin cancers [376]. Higher activity of Cat and SODs explains increased resistance of melanoma cells to oxidative stress compared to normal melanocytes and melanocytic nevi, suggesting that the acquisition of a robust antioxidant network is prominent for melanoma development [377,378]. A significant increase in plasma MDA and Cat activity but a simultaneous low SOD activity has been recorded in melanoma patients [379]. The elevation of both intracellular and extracellular ROS by tumor cells plays an important role in driving tumorigenesis by shaping the tumor microenvironment [380].

A particular feature of tumor stroma is the increased number of fibroblasts, pathologically activated and referred to as cancer-associated fibroblasts (CAFs). Due to the extraordinary tumor stroma paracrine dialogue, fibroblasts progressively acquire a molecular signature that partially overlaps with the stress-induced premature senescence one. This includes the release of molecules critically involved in metabolic and immune reprogramming of the tumor stroma with an effect on angiogenesis and resistance to therapy [381,382]. Extensive data demonstrated that ROS exerts a pivotal role in the process of fibroblast activation. It is known that ROS can be transferred from cancer cells to neighboring fibroblasts. ROS affects CAF’s features by promoting the conversion of fibroblasts to myofibroblasts that contribute to the tumor progression and spreading processes [383]. ROS activates CAFs that in turn enhance tumorigenesis by activating signaling pathways crucial for tumor cell proliferation and epithelial to mesenchymal transition [383]. As for other cancer types, the secretory profile of fibroblasts residing within the tumor margins or infiltrating melanoma substantially overlaps with that of senescent dermal fibroblasts [384,385]. It is important to underline that in contrast to the aging process that is characterized by a reduced number of dermal fibroblasts, skin neoplasms are permeated by the ratio of augmented fibroblasts/myofibroblasts, and that the presence of a large number of myofibroblasts in the tumor microenvironment has been associated with an elevated risk of invasion, metastasis, and a poor prognosis [364,386]. Paracrine TGFβ release by tumor cells causes an increase in NAD(P)H oxidase activity and triggers ROS-dependent fibroblast conversion into myofibroblasts by alpha-smooth muscle actin (α-SMA) gene transcription [387]. This crosstalk between tumor cells and CAFs could be abrogated with the addition of Cat [388]. Conversely, the decrease in oxidative stress in the tissue surrounding a tumor can slow down cancer growth and counteract its metastatic potential. Undeniably, SOD2 in CAFs can act as tumor suppressors [389].

## 6. Conclusions

Aging, as a broad term, encompasses several visible cutaneous phenomena such as skin wrinkling, atrophy, hair greying, and xerosis. Furthermore, since the skin gradually loses its structural and functional characteristics, skin aging is associated with several other processes such as viral, bacterial, and fungal infections, autoimmune blistering diseases, eczema, contact dermatitis, seborrheic dermatitis, and both melanoma and non-melanoma skin cancer. In this review, we provided an overview of the role of ROS in the appearance or the worsening of several age-related signs. We highlighted the importance of the former in both intrinsic and extrinsic aging. Indeed, chronic perturbation of redox equilibrium in the skin induces senescence and persistent inflammation. Overall, the understanding of the molecular mechanisms implicated in ROS formation and contrast is important to prevent physiologic skin aging and to compensate for functional deficits implicated in premature skin aging. However, the major challenging point is the definition of preventive strategies capable of sustaining the cutaneous intrinsic defense network. According to the minor capacity to preserve redox equilibrium, the elderly population needs to be more effectively treated to avoid an overabundance of ROS. On the other hand, pharmacological targeting of specific signaling pathways implicated in antioxidant/oxidant imbalance might represent a therapeutic opportunity for specific pathophysiological conditions frequently associated with aging, such as autoimmune disorders, chronic inflammation, and a multiple sequential skin cancer setting.
